# Localization of Metal Electrodes in the Intact Rat Brain Using Registration of 3D Microcomputed Tomography Images to a Magnetic Resonance Histology Atlas^1^,^2^,^3^

**DOI:** 10.1523/ENEURO.0017-15.2015

**Published:** 2015-07-22

**Authors:** Jana Schaich Borg, Mai-Anh Vu, Cristian Badea, Alexandra Badea, G. Allan Johnson, Kafui Dzirasa

**Affiliations:** 1 Department of Psychiatry and Behavioral Sciences, Duke University Medical Center, Durham, North Carolina 27710; 2 Department of Neurobiology, Duke University Medical Center, Durham, North Carolina 27710; 3 Center for Cognitive Neuroscience, Duke University Medical Center, Durham, North Carolina 27710; 4 Department of Radiology, Duke University Medical Center, Durham, North Carolina 27710; 5 Department of Biomedical Engineering, Duke University Medical Center, Durham, North Carolina 27710; 6 Department of Medical Physics, Duke University Medical Center, Durham, North Carolina 27710; 7 Department of Physics, Duke University Medical Center, Durham, North Carolina 27710; 8 Center for Neuroengineering, Duke University Medical Center, Durham, North Carolina 27710

**Keywords:** computerized tomography, electrode localization, magnetic resonance imaging, multielectrode physiology

## Abstract

Simultaneous neural recordings taken from multiple areas of the rodent brain are garnering growing interest because of the insight they can provide about spatially distributed neural circuitry. The promise of such recordings has inspired great progress in methods for surgically implanting large numbers of metal electrodes into intact rodent brains. However, methods for localizing the precise location of these electrodes have remained severely lacking. Traditional histological techniques that require slicing and staining of physical brain tissue are cumbersome and become increasingly impractical as the number of implanted electrodes increases. Here we solve these problems by describing a method that registers 3D computed tomography (CT) images of intact rat brains implanted with metal electrode bundles to a magnetic resonance imaging histology (MRH) atlas. Our method allows accurate visualization of each electrode bundle’s trajectory and location without removing the electrodes from the brain or surgically implanting external markers. In addition, unlike physical brain slices, once the 3D images of the electrode bundles and the MRH atlas are registered, it is possible to verify electrode placements from many angles by “reslicing” the images along different planes of view. Furthermore, our method can be fully automated and easily scaled to applications with large numbers of specimens. Our digital imaging approach to efficiently localizing metal electrodes offers a substantial addition to currently available methods, which, in turn, may help accelerate the rate at which insights are gleaned from rodent network neuroscience.

## Significance Statement

The digital imaging technique we present here allows unparalleled 3D visualization of the anatomical location of large numbers of electrodes implanted deep in the rodent brain. The anatomical location of each electrode can be determined quickly without removing the electrodes from the brain, can be observed from many different angles of view, and can be automated. These features offer substantial improvements over currently available histological methods of electrode location verification.

## Introduction

A major goal of neuroscience is to understand how spatially distributed neural networks facilitate specific types of behavior (Lewis et al., 2015). Rodents are ideal species for this application, because of their complex behaviors and suitability for genetic and molecular tools. To maximize the utility of using rodents to study spatially distributed neural networks, continuous neural activity must be assessed in many brain regions of a rodent brain simultaneously. Reflecting this notion, the United States’ Brain Research through Advancing Innovative Neurotechnologies (BRAIN) Initiative has labeled the measurement of multisite electrical activity in awake, behaving rodents a “high priority research area” (Advisory Committee to the NIH Director Interim Report, September 16, 2013).

The predominant method for measuring neural activity in rodents is to record electrical signals from 15- to 50-μm-thick metal microwires (electrodes) implanted into the brain. Thus, many efforts to measure neural activity across spatially distributed networks in rodents have used microwire multielectrode arrays (MEAs) to maximize the number of electrodes that can be implanted in one brain at the same time. Presently, recordings that use up to 64 microwires spread across up to five brain regions have been reported in rats (Nicolelis et al., 1997; de Araujo et al.,2006; Igarashi et al., 2014; Headley et al., 2015), and up to 64 microwires spread across up to 11 brain regions have been reported in mice (Sigurdsson et al., 2010; Dzirasa et al., 2011). In all of these cases, individual MEAs were implanted into spatially separated brain regions.

One of the methodological challenges of using MEAs is that although the techniques for implanting large numbers of electrodes have improved dramatically, the techniques for identifying the precise location of each electrode implanted in the brain have remained almost identical to those used throughout the last century. The most common way to determine electrode placements is to stain harvested brain slices with a nucleic acid stain (“Nissl stain”; Einarson, 1932), and infer the location of electrodes from the absence of stained neurons in a linear shape that matches that of an electrode. Although this electrode verification approach is effective for traditional applications, it is a very cumbersome and time-intensive procedure with disadvantages that become unmanageable when large numbers of electrodes are placed in the brain simultaneously. Most notably, brain tissue can be severely damaged before electrodes can be localized, electrode tracks can be altered by the removal of electrode bundles, and brain slices can be lost during the process of cutting or staining (Fischer et al., 2008). The normal wear and tear of occasionally losing or damaging a brain slice is not a problem for most applications, but it can become extremely disruptive when only one brain slice contains evidence of a particular electrode, as happens when electrodes are very thin (15-25 μm). Frustratingly for the MEA field, such problems are only exacerbated when the number of implanted electrodes increases, because closely packed electrode tracks decrease the integrity of brain tissue and make damage more likely. Since electrical recordings are rarely interpretable if their anatomical origins are unknown, these challenges of using histological stains to infer electrode placements have the potential to significantly limit the utility of multisite recordings.

Small animal brain imaging offers untapped strategies for solving the electrode location problems posed by multisite recordings. In theory, the most straightforward way to identify the exact location of implanted electrodes would be to image a brain while electrodes are still implanted. However, currently available imaging techniques, on their own, are not appropriate for this application. MRI is fatally susceptible to metal artifacts, including those caused by tungsten (the metal most commonly used to make electrodes) and the components in solder. MRI is also costly, and can require hours of scan time to produce an image with high enough resolution to identify very small brain regions. CT is an imaging modality that is less susceptible to metal artifacts, but provides close to no tissue differentiation in the brain under standard conditions. Therefore the ideal imaging solution would have the spatial resolution and tissue differentiation capability of MRI, the ability to visualize metal objects, and could be performed on intact animals with electrodes still implanted in their brains. Here we describe such a solution. Our method coregisters micro-CT images that depict the location of electrode bundles to a published high-resolution MR-atlas (Johnson et al., 2012). When completed, this method facilitates accurate anatomical localization of metal microwire bundles in a manner that is faster than any other method currently available.

## Methods

### Animal care and use

All animals were housed in a humidity- and temperature-controlled room, and water and food were provided *ad libitum.* Male Wistar Rats (*N* = 7; http://www.criver.com/products-services/basic-research/find-a-model/wistar-rat) were pair-housed and were implanted with recording electrodes at 6-7 weeks of age. All studies were performed in accordance with Duke University’s Institutional Animal Care and Use Committee’s regulations.

### Electrode implantations

Rats were anesthetized with isoflurane and placed in a stereotaxic frame. Stainless steel ground screws (Plastics One) were implanted above the anterior cranium and cerebellum; additional screws were implanted into the skull above the parietal lobe to improve stability. All screws were secured using C&B-MetaBond (Parkell). Craniotomies were made to allow implantation of the following S-isonel-coated tungsten arrays of 50 µm electrodes in the 10 areas (five areas bilaterally) listed in Table 1.

**Table 1.** Location of implanted tungsten electrode arrays

Brain region	Number of electrodes	Coordinates (mm from bregma and top of brain)		
		A/P	M/L	D/V
Bilateral anterior cingulate	2 per hemisphere	2.5	±.5	1.6
Bilateral orbitofrontal cortex	2 per hemisphere	3.7	±2	3.8
Bilateral anterior insula	4 per hemisphere	2.2	±4	4.5
Bilateral basolateral amygdala	4 per hemisphere	-3	±5.0	7.5
Bilateral olfactory amygdala	3 per hemisphere	-1.4	±3.2	8.8

**Figure 1:**
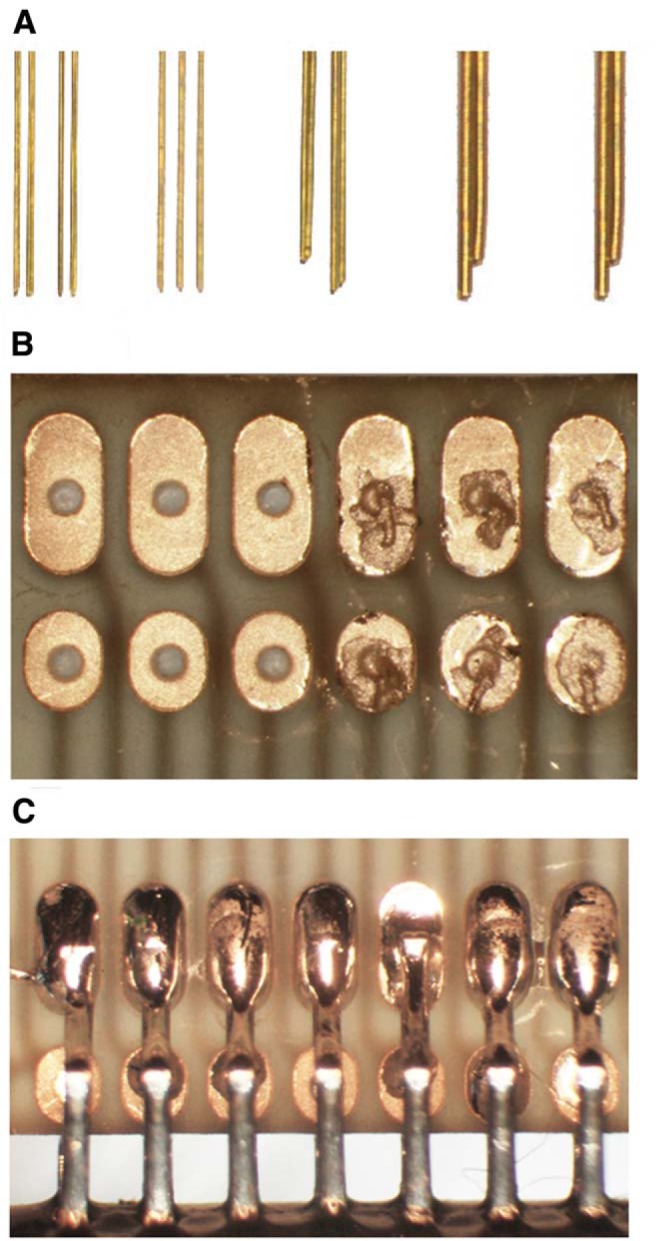
**Sources of metal in electrode implants. *A***, Tungsten electrodes. ***B***, Silver paint to connect tungsten wires to gold pads on the circuit board. ***C*,** Solder to connect the Omnetics connector to the circuit board.

The microwires in each array were separated by 250 µm. Each array was secured using additional C&B MetaBond. The tungsten electrode arrays (Fig. 1*A*) were connected to a printed circuit board using silver paint (Fig. 1*B*), and the circuit board was connected to a high-density miniature connector (Omnetics) using flux-cored solder (Fig. 1*C*; Kester SN63PB37 #66/44). The circuit board and connector were secured to the skull with dental cement after all electrode arrays were secured. All of the implant materials pictured in Figure 1 are commonly used in MEA preparations, but prevent the acquisition of MR images and cause artifacts in micro-CT images.

### Micro-CT scanning

Rats (*N* = 7) were anesthetized with pentobarbital (250 mg/kg) and perfused transcardially with 200 ml PBS, pH 7.4, followed by 200 ml 4% paraformaldehyde in PBS. Intact heads were then detached from the body and refrigerated until scanning. Image data were acquired with a dual-source micro-CT system. The system contains two G-297 x-ray tubes (Varian Medical Systems) with 0.3/0.8 mm focal spot size, two Epsilon High Frequency x-ray generators by EMD Technologies, and two CCD-based detectors with a Gd2O2S phosphor (XDI-VHR 2 Photonic Science) with 22 μm pixels binned to 88 μm. The data acquisition was controlled by sequencing applications written in LabVIEW. Only one of the x-ray sources/detectors was used for these scans. Three hundred and sixty projections were acquired at 140 kVp, 50 mA, and 5 ms per exposure with Cu filtration. Tomographic reconstruction was performed using the commercial software COBRA EXXIM (Exxim Computing; http://www.exxim-cc.com/products_cobra.htm). Metal causes beam hardening and scatter effects in CT images, which result in dark streaks between metal objects and bright streaks in the surrounding area. COBRA EXXIM provided a proprietary iterative streak and metal artifact-reduction algorithm (SAMARA) that is more tolerant to data imperfections than the standard Feldkamp method (Feldkamp et al., 1984). Artifacts are often handled by using an intensity cut-off to remove the pixels comprising the artifact, and replacing the missing voxels using interpolation of the surrounding voxels. Consequently, voxels close to the object causing the artifact are often “smoothed out” and details are lost. The SAMARA algorithm removes artifacts while maintaining low and medium contrast around the artifact-causing object. The SAMARA parameters were set such that streak artifact reduction was performed if SAMARATAG_HIGHCONTRASTLEVEL= 1000 and SAMARATAG_LOWCONTRASTLEVEL= 0. Metal artifact reduction was also performed if the parameter SAMARATAG_HIDENSLEVEL = 15000. The volumes were reconstructed in a 640^3^ matrix with a voxel size of 88 μm.

### Immunohistochemistry

After CT scanning, the upper half of each rat's skull was removed with the electrodes still attached, and the perfused brains were extracted from the rest of the skull. The extracted brains were then postfixed overnight in 4% paraformaldehyde at 4°C, put in 30% sucrose at 4°C until they sank (2-3 days), flash-frozen in isopentane, and stored at −80°C. Each brain was sectioned into 30 μm slices on a cryostat (Leica Microsystems) and mounted onto gelatin-subbed slides. All sections were processed using a colorimetric Nissl stain. For the Nissl stain, mounted slides were placed in a 1:1 chloroform/alcohol mixture for 1 h, gradually rehydrated through increasing concentrations of water:alcohol, stained with cresyl violet, cleared with an alcohol + glacial acetic acid, gradually dehydrated through decreasing concentrations of water:alcohol followed by xylene, and coverslipped using Permount Mounting Medium. Images of stained slices were taken with a Zeiss Axio Observer Z1 microscope at 10×magnification.

### CT-to-MR atlas registration

The overall goal of the registration process was to register CT images of individual rats implanted with electrodes to a previously published multidimensional magnetic resonance histology (MRH) atlas of the adult Wistar rat brain (Johnson et al., 2012). This process differs from previously published CT-MR registration procedures that register CT images of an individual to MR images of that same individual (Maintz et al., 1996; Vaquero et al., 2001; Princich et al., 2013; Azarion et al., 2014; Mirzadeh et al., 2014), or registration procedures that register MR images of one individual to a canonical MR atlas (Sergejeva et al., 2015). Since metal artifacts prevented the acquisition of MR images from implanted rats, the registration process for the present application had to both cross modalities (CT to MR) and individual specimens (an individual CT scan to an atlas representing the average of five other specimens).

Procedures involved in image registration have been described and reviewed extensively elsewhere and will not be detailed here (Gholipour et al., 2007). At their core, these procedures aim to compare two image volumes iteratively until their “similarity” is maximized. Registration procedures have three main components: transformation specifications, which define what kinds of degrees of freedom are allowed; a measure of similarity between the images aligned; and an optimization framework, which determines how possible transformation parameters will be chosen. When registration procedures fail, they usually do it in one of two ways. The first kind of failure is when images are dramatically misaligned. This often occurs because the optimization strategy did not lead to an adequate sampling of the possible model parameters before converging on a solution, leading to transformation parameters that represent a local solution to the registration problem rather than a global solution. The second type of failure occurs when images are aligned approximately in the correct space, but features of interest do not line up satisfactorily. This usually occurs because images were not transformed in the correct dimensions or with enough degrees of freedom. The registration procedure described below minimized both types of errors. The code used to implement these procedures can be found at http://www.civm.duhs.duke.edu/eNeuro2015/.

**Figure 2:**
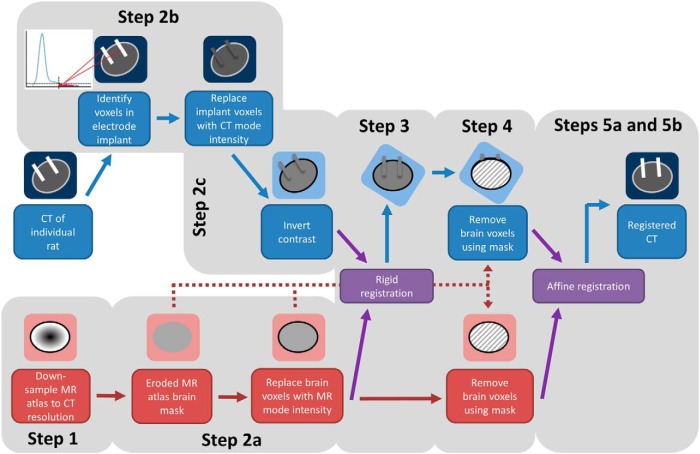
Schematic of micro-CT to MRH registration procedure.

Since soft tissue is poorly resolved in CT images, our registration approach was based on the skull. We found that two general strategies improved the success of this skull-focused approach. First, although nonrigid registration transformations were necessary to account for the differences in sizes and shapes between individual rats and the rat atlas, nonrigid registration was greatly improved when it was preceded by a rigid registration step to place the CT images and MRH atlas in approximately the correct space. Second, registration was improved when as much of the nonskull image as possible was masked out of the CT images and MR atlas. Implementing these steps drastically reduces the likelihood of converging on transformation parameters that represent nonglobal registration solutions. These strategies resulted in the multistep registration procedure described below and illustrated in Figure 2. Although the general approach behind this procedure should work for most scanning setups, specific parameters may need to be optimized for different CT scanners, scanning parameters, or electrode implantation methods.

#### Step 1

The acquisition methods and processing technique used for the MRH atlas were described in detail by the group that published the atlas (Johnson et al., 2012). The MRH atlas was downsampled to match the 88 μm isotropic voxel size of the CT images. (If a CT system with a different voxel size is used, the MRH atlas should be downsampled to match the size of CT data or the CT images should be downsampled to match the 25 μm voxel-size of the T_2_*-weighted structural images of the MRH atlas. Take note that the diffusion-weighted image series of the MRH atlas is published at a resolution of 50 μm.)

#### Step 2

The MRH atlas and CT images were preprocessed to prepare them for a rigid registration step that would arrange them into approximately the same space.

#### Step 2a

The goal of this step is to mask out brain tissue differentiation from the chosen MRH image. The MRH atlas consists of a number of different datasets, each of which emphasizes a different type of soft tissue contrast. All the datasets are registered to the same space, so registration of the CT data to one dataset effectively registers the CT data to all of the MR atlas images. Because of the inclusion of a skull-stripped version of the image, we chose to register the CT data to the “b0 image,” which is a nondiffusion-weighted spin echo image that provides the baseline for diffusion tensor data acquisition. To do so, we made a “brain mask” using the boundaries of the skull-stripped b0 image. This initial brain mask was eroded with a sphere 20 voxels in diameter to ensure that no skull would be masked out. Voxels falling within the brain mask were then replaced with the mode intensity value of the MRH image. This mask resulted in an MRH image with homogeneous intensity values within the skull.

#### Step 2b

The goal of this step is to mask the electrode implant (referring to the electrodes, circuit board, wires, and connector) out of the CT image and replace the masked voxels with the mode intensity value of the CT image. The method we used to isolate the electrode implant from the CT images in our preparation was based on the observation that voxels representing the electrode implant had higher intensity values than anything else in the image (including the skull) and appeared with a very low probability. This electrode feature also has been found in CT images of human deep brain stimulators (Azarion et al., 2014). Taking advantage of these features, the most straightforward way to mask out the electrode implant was to estimate a probability density function of the intensity values in each of the CT image volumes. The estimate of this function was computed using the ksdensity function in MATLAB R2013 (The MathWorks), which is based on a normal kernel function and is evaluated at 100 equally spaced points that cover the full range of the inputted data. All voxels that had intensity values greater than the mean intensity value and an estimated probability density of <0.00001 were part of the electrode implant. This electrode implant footprint was then dilated by a sphere five voxels in diameter to ensure complete coverage of the electrodes and implant, and all the voxels on an individual CT scan within this final mask were replaced with the mode intensity value of that individual CT scan. This mask resulted in a CT image with effectively no electrodes or head implants. Other strategies for masking out electrodes can be used as necessary for specific preparations; the critical part of Step 2a is that voxels representing the electrode implant are replaced with the mode intensity value of the CT image.

#### Step 2c

The intensity values of the CT images were inverted so that skull would have very low intensity values (would look dark to the eye) compared with the rest of the image, similar to the skull on the MR b0 image.

#### Step 3

The CT images were registered to the MRH b0 atlas using a rigid registration transformation calculated by the imregister function in MATLAB R2013 (The MathWorks). As stated earlier, the goal of this step was to place the CT and MR atlas images in approximately the same space to optimize subsequent nonrigid transformations performed in later steps. Mattes Mutual Information—a statistical measure from Information Theory indicating the amount of information one random variable (image) gives about another (Mattes et al., 2001)—was used as the similarity metric for this step because of its established success in multimodal imaging applications (Pluim et al., 2003). The One Plus One Evolutionary optimizer works by generating random samples around the current position in parametric space (Styner et al., 2000); we found this optimizer reduced the number of dramatic registration failures from nonglobal transformation solutions. The final parameters were as follows: initial radius = 1.01, growth factor = 0.00325, maximum iterations = 500, optimizer = OnePlusOneEvolutionary, metric = MattesMutualInformation.

#### Step 4

The MRH atlas and CT images were preprocessed to prepare them for affine (nonrigid) registration. To minimize registration interference from the brain tissue within the MRH b0 image, the brain mask from Step 2a was eroded with a sphere five voxels in diameter and applied to both the rigidly registered CT and the b0 image. In this case, we found registration to be best if voxels were fully excluded from the registration process rather than be replaced by mode intensity values.

#### Step 5

The rigidly registered CT images were registered to the MRH b0 atlas using a nine parameter affine similarity registration transformation, allowing for translation, rotation, and scaling to improve the overall fit due to anatomical differences between the specimens and the MRH atlas.

#### Step 5a

The similarity registration transformation was calculated using the imregister function in MATLAB (The MathWorks). As in Step 2c, Mattes Mutual Information was used as the similarity metric and the One Plus One Evolutionary was used as the optimizer. The final parameters were as follows: initial radius = 1.01, growth factor = 0.00325, maximum iterations = 500, optimizer = OnePlusOneEvolutionary, metric = MattesMutualInformation.

#### Step 5b

The resulting rigid and affine transformation matrices were recorded and applied to the original, unmasked CT images.

### Assessment of registration success

Perfect registration between CT images and the MRH atlas would lead to perfect alignment of all corresponding points in both images. Assessing the accuracy of this alignment would therefore require measuring the distance between a very dense set of landmarks present in both sets of images that were not used in the registration process. Like many digital imaging situations, such landmarks were not available. Surrogate measures have been proposed to help assess the accuracy of image registrations, including tissue label overlap scores and image similarity measures, but recent studies have shown these measures to be unreliable (Rohlfing, 2012). Thus, expert knowledge and evaluation is considered the gold standard of image registration evaluation (Akbarzadeh et al., 2013).

For the present study, this expert validation was provided in the form of three human rat anatomy experts who assessed through visual inspection (1) the overlap of skull features in the CT images and MRH images and (2) the correspondence of electrode bundles in the registered CT overlaid on the MRH atlas with the physical electrode tracks observed in physical slices of the same brain. Examining the overlap of skull features is accomplished by overlaying the registered CT images and MRH images in an image visualization package such as the freely available 3D Slicer (www.slicer.org; Fedorov et al., 2012) and toggling through different transparency levels of the two image volumes. An anatomy expert familiar with the image visualization software can accomplish this type of visual inspection in about 1 min.

The correspondence of electrodes in the registered CT with the physical electrode tracks observed in physical brain slices was examined by comparing CT images overlaid on the MRH atlas with collected histological brain slices viewed under 4× magnification. Distances were measured on the CT overlaid on the MRH atlas using the ruler tool in 3D Slicer. Distances were measured on the histological slices using the ruler tool in the Nikon NIS-Elements Basic Research Microscope Imaging Software (version 3.2) calibrated to a Nikon Plan Fluor 4×/0.13 microscope lens. Experts chose 15 electrodes and measured distances from an electrode to a landmark in the brain in both the registered CT overlaid on the MRH atlas and in the histological slice. The distances were then compared by subtracting the distance measurements from one another. The median of all the difference measurements was used to estimate the accuracy of the CT-to-MRH method. Each expert chose their own electrodes and their own landmarks.

### Electrode configuration simulations

The goal of the electrode simulations was to determine whether the configuration of electrode bundles would affect the consistency and robustness of our registration procedure. To accomplish this, first, we acquired a CT scan of one nonimplanted rat. The CT scan was registered to the MRH atlas as described above (except no masking of electrodes was required), and the rigid and affine transformation matrices were saved. Next, a set of 1000 fiducials was randomly generated and applied to the nonregistered CT image of the nonimplanted rat. These fiducials provided a dense set of landmarks present in both sets of images that could be used to evaluate the change in the registration process caused by the addition of simulated electrodes. Twenty-three fiducials falling outside of the skull (as determined by back-transforming the MRH brain mask to the native space of the CT image) were discarded, leaving a total of 977 fiducials. The coordinates of the 977 fiducials were then transformed using the saved rigid and affine registration transformation matrices, and these newly registered coordinates were used as the reference coordinates for all subsequent electrode simulations.

Third, we simulated 1000 implanted rat skulls. To simulate different electrode configurations, the electrode bundles and the rest of the head implants were isolated using voxel intensity values from the registered CT image of each implanted rat. For each simulated electrode configuration, one isolated head implant and 10 electrode bundles were randomly chosen to be applied to the CT image of the nonimplanted rat. The head implant was shifted by random amounts along the anterior–posterior and left–right axes. The electrode bundles were shifted by random amounts along the anterior–posterior, left–right, and dorsal–ventral axes. The amount of translation for each implant or bundle along each axis was determined by independent random draws from Gaussian distributions with a mean of 0 and an SD of five voxels, with the following exception: to allow for even more variation in simulated wire placement, the amount of translation for the bundles along the anterior–posterior and left–right axes was drawn from a Gaussian distribution with a mean of 0 and an SD of 10 voxels. To make sure simulated electrode bundles did not extend outside of the skull, all electrodes that extended outside of the MRH brain mask were removed. The resulting simulated head implant and collection of electrode bundles were then back-transformed to the native space of the nonimplanted CT, and placed on the nonimplanted CT image by replacing the CT values in those voxels with the intensity values of the original head implant or electrode bundle.

Each simulated CT image was registered using the procedures described for the CT images of the truly implanted rats (all stated parameters were the same, except the brain mask for the rigid registration was eroded with a 10 voxel sphere instead of 20 voxel sphere). For each of the 1000 simulations, the new set of registration transformation matrices acquired through the registration process was applied to the unregistered set of 977 fiducials. The coordinates of these fiducials were compared with the coordinates recorded from the registration of the nonimplanted rat. The Euclidean distance between each fiducial and its corresponding fiducial on the reference list was calculated after the rigid registration step and again after the nonrigid registration step. For each simulation, the mean, SD, and median of the distance (or “movement”) between the simulated and reference coordinates across all 977 fiducials were recorded. Estimates of the registration error caused by the head implant and electrodes were assessed by analyzing these distance metrics of the 977 fiducials in each of the 1000 simulations (977,000 fiducials in total).

### Statistics used to evaluate electrode configuration simulations

When statistical tests are discussed in the main manuscript, they will be followed by a superscript lowercase letter. This letter corresponds to the lower case letter preceding each row of the “Goal” column in Table 2.

**Table 2.** Goal and characteristics of statistical tests used to evaluate electrode configuration simulations

Goal	Distribution of data	Type of test	Power of test
^a^Test differences in fiducial movement between brain segments after rigid registration only	Highly skewed right	Kruskal–Wallis	χ^2^ = 3.00e+05
^b^Test differences in fiducial movement between brain segments after rigid registration followed by nonrigid registration	Highly skewed right	Kruskal–Wallis	χ^2^ = 2.33e+05
^c^Test differences in fiducial movement after rigid registration and rigid registration followed by nonrigid registration	Highly skewed right	Mann–Whitney *U*	*Z* value = 30.4165 Rank sum = 1.01e+12

## Results

**Figure 3:**
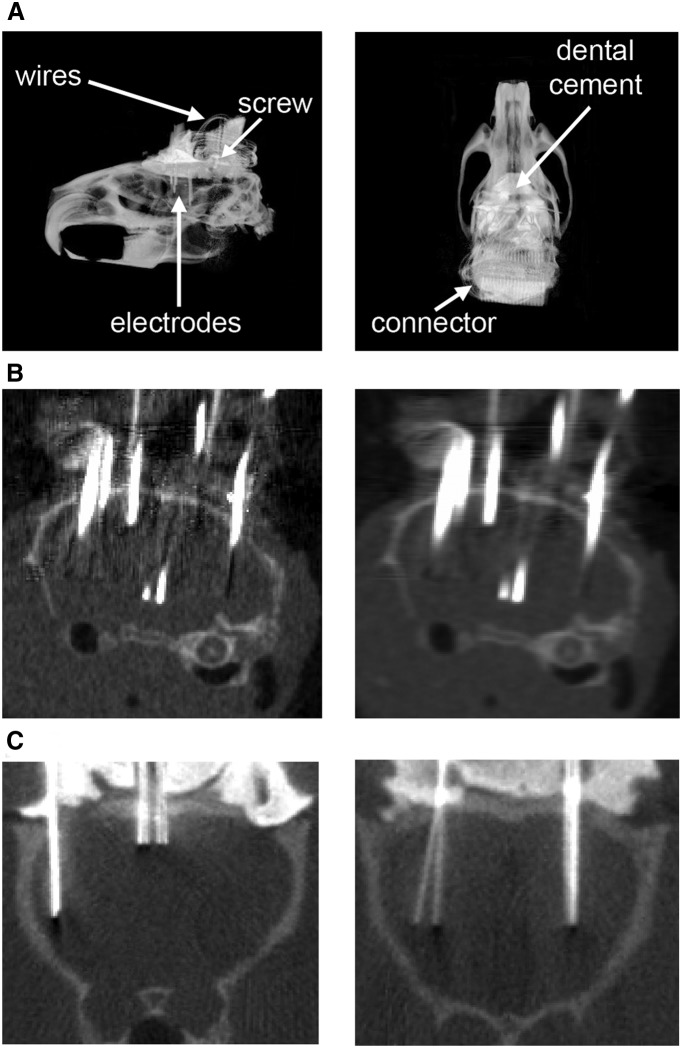
**Micro-CT images of implanted rat brains. *A*,** 3D reconstructions illustrate 3D structure of electrode bundles and head implants. ***B*,** Examples of CT cross sections without (left) and with (right) artifact reduction applied. ***C*,** Eighty-eight micrometer voxels do not permit clear differentiation of individual wires within electrode bundles (2-4 individual wires are contained in each pictured bundle, despite their appearance as singular objects), but separate bundles are easily visualized (left), as are individual wires that are severely misplaced or bent (right).

The CT scans successfully allowed visualization of both the skull and the implanted electrode bundles (Fig. 3*A*). Although the metal in the skull screws and head implant did cause considerable image artifacts, artifact reduction procedures significantly improved the homogeneity of the images (Fig. 3*B*). The 88 μm voxel size of our micro-CT system could not always clearly differentiate between individual microwires within a correctly implanted bundle (Fig. 3*C*, left; differentiation between microwires in the insula bundle on the left side of the brain is less clear, differentiation between microwires in each midline anterior cingulate bundle is more clear), but it did allow visualization of individual, severely misplaced or bent microwires (Fig. 3*C*, right), as well as bundles separated by more than 250 µm (Fig. 3*C*, right; anterior cingulate bundles at midline are separated by 500 µm). As expected, no brain tissue could be differentiated from CT scans.

**Figure 4:**
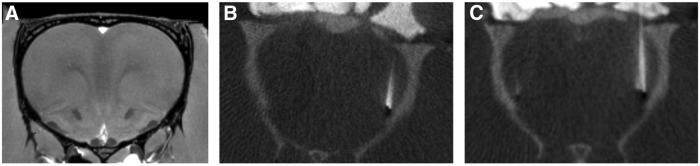
**Example of registration between individual CT scans and the MRH atlas. *A***, Coronal slice of MRH atlas. ***B***, Corresponding slice in unregistered CT. ***C*,** Corresponding slice in registered CT.

**Figure 5:**
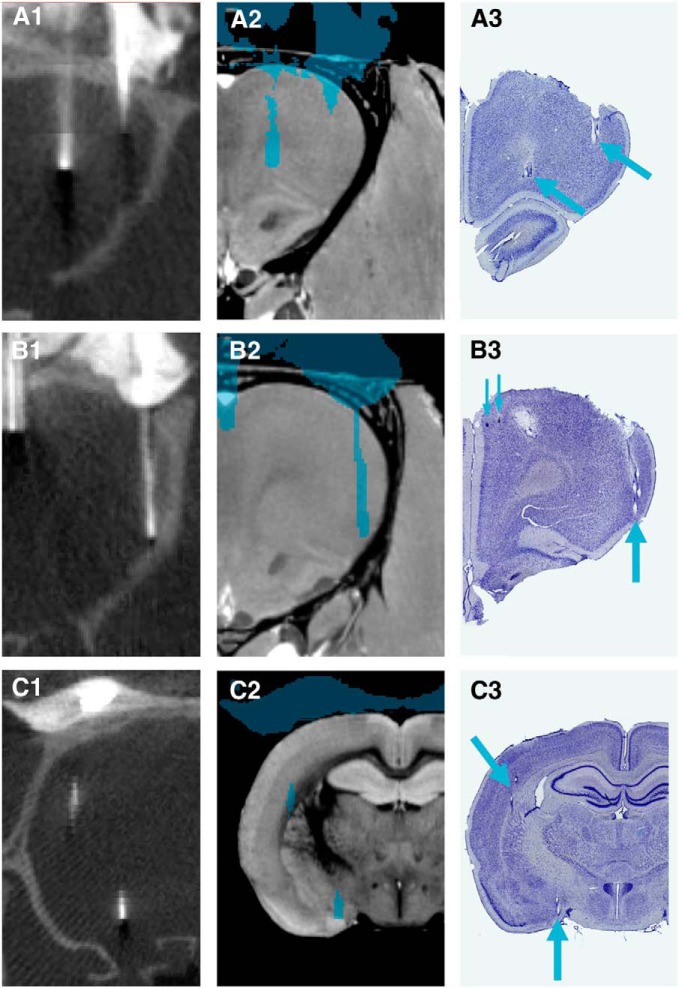
**Histological validation of CT to MRH atlas registration.** Column 1, CT image. Column 2, Registered electrode mask from CT overlaid on MRH atlas. Column 3, Corresponding brain slice stained with cresyl violet. Row A, Orbitofrontal cortex bundle and top of insula bundle (both in correct location) overlaid on the b0 image from the MRH atlas. Row B, Anterior insula bundle (correct location) overlaid on the b0 image from the MRH atlas. Row C, Olfactory amygdala bundle (landed on the very lateral edge of intended area) overlaid on the gradient recalled echo image from the MRH atlas. In each row, the atlas contrast image that best highlighted the soft tissue architecture of the brain area around the electrode was chosen. For the examples illustrated in Rows A and C, the physical brain slices used for histology were not cut perfectly perpendicular to the anterior–posterior axis. Highlighting the advantages of 3D image volumes, the registered CT overlaid on the MRH was resliced to match the plane of the histology slice shown in Column 3 (as illustrated).

Our multistep registration procedure based on the skull yielded convincing registration between individual CT scans and the MRH atlas. Three anatomy experts reported excellent correspondence (less than 0.5 mm in all locations) between the boundaries of the CT skull and those of the MRH atlas skull in all seven rats (Fig. 4). Confirming this correspondence, the histology performed on brain slices after CT scanning indicated that the coregistration procedure yielded very accurate results in all animals (examples shown in Fig. 5). Highlighting one of the advantages of using a 3D MRH atlas, when the physical brain slices used for histology were not cut perfectly perpendicular to the anterior–posterior axis, the registered CT overlaid on the MRH could be “resliced” to match the plane of the histology slice without losing any image resolution. The anatomy experts estimated a median difference of 32 µm between the location of the electrodes in the registered CT and the location of electrode tracks in stained brain slices.

**Figure 6:**
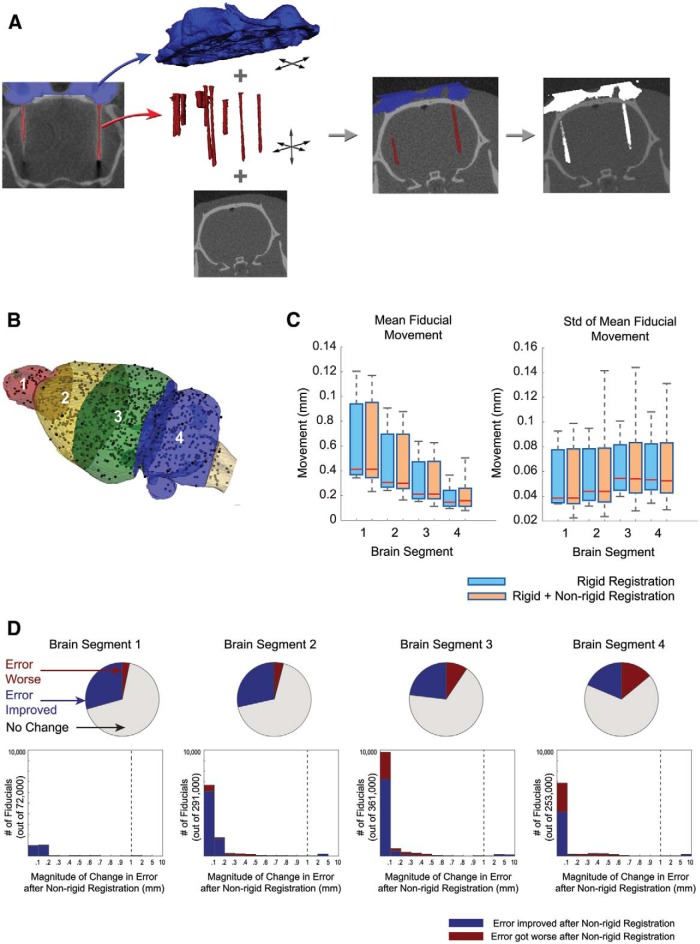
**Results of electrode and head implant simulations. *A***, Schematic of simulations. ***B***, Illustration of 977 fiducials on a glass brain separated into four labeled segments. ***C***, Box plots of the mean and SD of Euclidean distance between all fiducials on the registered (after both rigid and nonrigid registration) nonimplanted brain and registered, simulated implanted brains across all 1000 simulations. Red line, median of all 1000 simulations. Box limits represent the 1st and 3rd quartiles. Whiskers represent the 5th and 95th percentiles. ***D***, Percentage of 977,000 fiducials (the 977 fiducials that landed within the confines of the brain across 1000 simulations) whose Euclidean distance between the registered simulated implanted brain and nonimplanted brain was reduced, increased, or unchanged by the nonrigid registration step compared with rigid registration alone (pie charts). Histograms of the absolute value of the change in error (in Euclidean distance) caused by the nonrigid registration step (stacked bar charts; unchanged fiducials excluded) in the 977,000 fiducials across all imputations. Note that bin sizes increase after the dashed line.

Simulation experiments illustrated that head implants and electrode bundles did affect the registration process (Fig. 6*A–D*). The median change in fiducial coordinates when implants and electrodes were present compared with when they were absent was ∼0.2-0.4 mm, depending on the brain location (Fig. 6*C*). Fiducials in anterior parts of the brain were more affected by the presence of electrodes than fiducials in posterior parts of the brain (Fig. 6*C*; significant differences between brain segments after rigid registration, as well as after rigid plus nonrigid registration, *p* < 0.001^a,b^, Kruskal–Wallis test). The latter result is likely because the orientation of the olfactory bulb and most anterior parts of the frontal cortex relative to surrounding bone structures are extremely variable from animal to animal, whereas the orientation of the center of the brain is much more consistent. Of note, the nonrigid registration step did not dramatically change fiducial placements within these simulation experiments (Fig. 6*D*). When it did change the fiducial placements, however, on average it placed them closer to the coordinates retrieved from registration without simulated implants and electrodes more often than it placed them farther away. Applying nonrigid registration after rigid registration resulted in a median change of 0.26 mm from the coordinates retrieved from registration without simulated implants, whereas applying rigid registration alone resulted in a median change of 0.27 mm from the coordinates retrieved from registration without simulated implants (*p* < 0.001^c^, Mann–Whitney *U* test).

Still, sometimes the nonrigid registration step did make the registration worse as assessed by fiducials landing farther away from the coordinates retrieved from registration without simulated implants after rigid and nonrigid registration was applied than when only rigid registration was applied (Fig. 6*D*). These detrimental effects, when they occurred, were more likely to manifest in posterior parts of the brain than anterior parts of the brain (Fig. 6*D*, pie charts), but were usually small in magnitude (<0.1 mm, shown by the red bars in the histograms of Fig. 6*D*). Occasionally registration failed dramatically before or after the nonrigid registration step during these simulations (as reflected by the tails of the histograms in Fig. 6*D*), due to the selection of transformation parameters that optimized local solutions rather than global solutions. When these failures occurred, they were visually obvious and resulted in significant lack of overlap between the CT image volume and the MRH atlas, so they could easily be detected. Once detected, we found that these failures could be corrected by tuning the mask and registration parameters.

## Discussion

Measuring electrical activity from electrodes implanted across multiple brain areas simultaneously in awake, behaving rodents holds great potential for uncovering the mechanisms underlying distributed neural networks. Multisite recording technology has made significant strides. The next step is to implement electrode localization methods that permit these exciting recording technologies to reach their full potential.

Here, we have described a technique that combines micro-CT images with a high-resolution MRH atlas to identify the anatomical location of metal electrode bundles in an intact rodent brain. The 3D view of all implanted electrodes provided by our technique provides an unparalleled opportunity to visualize the true trajectory of each electrode bundle and measure the spatial relationship between multiple independent bundles *in vivo*. Our technique does not require removing the electrodes from the brain, nor does it require the surgical implantation of precisely placed external markers. Furthermore, unlike physical brain slices, the 3D images of the electrode bundles and the MRH atlas they are registered into can be resliced to view any desired plane of tissue without extra manual work or expensive reconstruction software (Markovitz et al., 2012). This allows for the verification of the location of electrode tips from many different visual angles with ease. In addition, the MRH atlas contains eight different interchangeable reference images that use unique contrasts to highlight specific aspects of soft tissue architecture; having access to the entire collection of images improves opportunities to visualize the precise anatomical location of the end of an electrode. Although the preparation used in the current study used perfused animals, this technique could easily be extended to live anesthetized animals as well.

The primary alternative to using our digital imaging approach for localizing electrodes is to use standard histological (Nissl) techniques. Histological techniques have severe limitations that become increasingly problematic as the number of electrodes implanted in the brain grows. The first limitation is that histological techniques require a significant time investment. After a brain is removed from a skull, it must be postfixed, cryoprotected in a sucrose solution overnight, and flash frozen before it is ready to slice. Then, very thin slices are painstakingly cut one by one and mounted onto slides for staining and eventually cover slipping (Gerfen, 2003). Overall, the entire electrode localization procedure takes days to weeks to complete if multiple brains must be processed sequentially. In comparison, our imaging technique takes 15-20 min after the brain scans are acquired. This improvement in speed is one of the greatest advantages of our digital imaging approach.

A more problematic, but less ubiquitous, limitation with histological techniques are that slice preparation and Nissl stain protocols can severely damage critical brain tissue before electrodes can be localized. Especially when strong adhesives and multiple screws are needed to secure electrical connectors to the skull of an animal, the process of removing electrode bundles from a brain can bend the electrodes, alter the electrode paths, or cause extra tissue damage. Cutting brain slices is also a notoriously finicky process that can result in the loss of brain slices. Furthermore, traditional Nissl stains require many dehydration and hydration steps that may warp and damage brain tissue. Even if the tissue processing is completely perfect, it is often difficult to identify the precise location of the tip of very thin electrodes, especially if brain slices are not cut in the exact same geometric plane as the electrode tracks. All of these limitations can be overcome or completely avoided using our CT-to-MRH atlas digital imaging technique.

**Figure 7:**
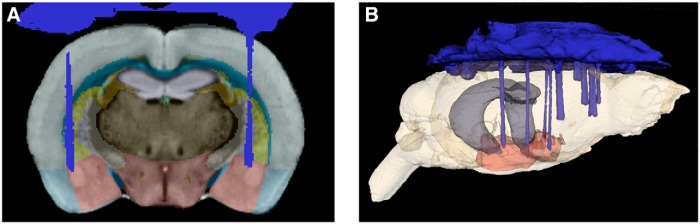
**Electrode bundles overlaid on segmentation atlas. *A***, Coronal brain slice of two amygdala bundles overlaid on the segmentation atlas and gradient recalled echo atlas image. The colors of the segmentation atlas are as follows: orange, amygdala; purple, hippocampus; yellow, caudate/putamen; blue, corpus callosum. ***B***, 3D rendering of all bundles overlaid on a glass brain with the amygdala (orange) and hippocampus (purple) from the segmentation atlas shown.

One of the greatest benefits of our CT-to-MRH atlas technique that would be difficult and potentially cost-prohibitive to accomplish using histology alone is that localizing metal electrodes can be fully automated once CT images are acquired. Consequently, metal electrodes can be localized in large numbers of specimens without much manual labor. Furthermore, as atlases become increasingly detailed the utility and programmability of the method will increase. In particular, it will become possible to automate the final verdict of whether a particular electrode bundle landed in an intended anatomical location. Figure 7 illustrates how a currently available segmentation atlas makes it possible to determine whether bundles in the present study landed in the amygdala; once a sufficiently segmented rat atlas becomes available with the boundaries of all brain regions demarcated and labeled, it will be possible to determine whether electrodes landed in even smaller brain structures or specific nuclei.

Another feature of our approach is that the success of any registration can be confirmed by a visual inspection that assesses whether the overlap between the skull of the CT image and the skull of the MRH image is satisfactory. An automatic algorithm can be used to detect dramatic registration errors, but we recommend making specific efforts to visually confirm registration results when multiple electrodes are implanted in the most anterior parts of the brain, because our simulation experiments indicated such electrode placements are most likely to interfere with registration. Such visual inspections should take no more than a few minutes per rat. If a particular registration is found to be suboptimal either by an automatic algorithm or visual inspection, users can tune the mask and registration parameters until the overlap between the CT and MRH skulls is acceptable. When tuning is required, we recommend varying the initial radius and growth factor registration parameters rather than changing the optimizer or similarity metric.

In future applications, CT systems using smaller voxel sizes can be used to aid in visualizing very thin electrodes or localizing electrodes to small nuclei. The custom-built system used in the present study was optimized for other *in vivo* micro-CT applications where the resolution of 88 µm is adequate. This level of resolution is informative for localizing 50 µm electrodes to larger structures, such as the amygdala, that are at least 1000 µm in width and height. When higher resolution localization is necessary, commercial systems with resolutions as small as 4.5 µm can be used (e.g., the Quantum GX microCT Imaging System by PerkinElmer), although the radiation dose required for this level of resolution necessitates terminal or postmortem studies. Importantly, the general procedure for registering CT images collected at <80 µm resolution to the MRH atlas would be the same as those described here (with the possible addition of an extra downsampling step if the CT resolution is smaller than that of the MRH atlas, followed by a transformation step to the CT images at their original resolution). Also useful to remember is that no matter at what resolution the CT images are collected, they will be overlaid on the MRH atlas that has a resolution of 25 µm.

The present paper focuses on applying the CT-to-MRH atlas electrode localization technique to microwire MEAs because they often contain more metal than other types of MEAs and are therefore particularly challenging to visualize using metal-sensitive imaging techniques such as MRI. Another interesting future direction for the work presented here, perhaps in combination with using CT imaging protocols that allow higher resolution, is to extend the CT-to-MRH atlas to other types of MEAs, such as multisite silicon probes. Silicon probes are MEAs with multiple flat, metallic recording sites spaced along nonmetallic probes (in contrast with the microwire MEAs described in the present study, which are completely metal and have recording sites restricted to the end of the electrodes). It is now possible to use multishank, high-density recording silicon probes to record up to 512 channels in awake, behaving rodents (Berényi et al., 2014). Just as with multiwire MEAs, precise anatomical localization of each recording site on a silicon probe is important for interpreting acquired neurophysiological data.

Based on the results we describe here, the CT-to-MRH atlas electrode localization technique is a promising option for localizing silicon probe recording sites efficiently and cost-effectively. To assess this option, the first step would be to determine whether either the outline of the silicon probe implants or the recording sites themselves could be visualized using CT imaging. If the proportion of x rays that pass through the material used to make the silicon probe differ from the proportion of x rays that passes through brain tissue, the outline of the silicone probe should be visible using CT. The likelihood of successfully visualizing individual recording sites will depend on the type of metal used, but given that the wire MEAs used in the present study contained more metal than most silicon probe preparations, we do not anticipate that metallic artifacts caused by silicon probes will be debilitating. Assuming that either the probe outline or the recording sites can be visualized using CT, the next step would be to tailor the technique to the specific preparations and goals of each experiment. Silicon probe designs vary widely (see http://neuronexus.com/products/neural-probes and http://www.cambridgeneurotech.com/ for commercial examples), but the probes are typically 15 μm thick, and have heterogeneous widths that often begin with a sharp tapered tip and grow to around 100 μm at the uppermost recording site. The metal recording sites may range in area from 150 to 300 μm^2^, and are spaced anywhere from 20 to 200 μm apart, depending on the application. As an example of how individual probe designs may affect how the CT-to-MRH atlas would be applied, if recording sites are spaced 20 μm apart and the study goals require each recording site to be visualized individually, electrode localization steps will likely need to be implemented postmortem with a CT system that can accommodate small voxel sizes of <10 μm. However, if the recording sites are spaced 200 μm apart, larger voxel sizes compatible with nonlethal radiation doses will likely be sufficient. Furthermore, since the distance and geometric configuration of recording sites in silicon probes is known with machine-implemented precision, the anatomical location of each recording site can be determined as long as the boundaries and/or axis of the probe are known, and larger voxel sizes may be sufficient to visualize these properties of the probe. More research needs to be done to determine how to best optimize CT imaging of silicon probes, but extending the CT-to-MRH atlas method to other types of MEAs will be a fruitful area for future development.

The CT-to-MRH atlas approach to efficiently localizing metal electrodes we describe here offers a substantial addition to currently available histological electrode placement strategies in multiple domains. Our general registration strategy can be easily adapted to new CT imaging and electrode implant procedures. The field of neuroscience is in a very exciting time where it is now possible to measure electrical activity in multiple areas of the rodent brain at the same time. Taking advantage of the power of digital imaging to advance efficient electrode localization strategies will, in turn, accelerate the rate at which these new multisite recording techniques will help to decipher the mechanisms and functions of spatially distributed neural networks.

## Synthesis

The decision was a result of the Reviewing Editor Gyorgy Buzsaki and the peer reviewers coming together and discussing their recommendations until a consensus was reached. A fact-based synthesis statement explaining their decision and outlining what is needed to prepare a revision is listed below. The following reviewer(s) agreed to reveal their identity: Santiago Canals

### Synthesis of Reviews:

Registering the physiological signals with the underlying anatomical circuits which gave rise to them an important issue. This work describes a method to register CT image of individual rat brains to MR rat brain atlas. This method can be used for localizing lesions, electrodes, infusion devices, etc mostly in case of multiple electrodes implanted in the rodent brain. The major advantage of the proposed technique is its speed and perhaps low cost, compared to high resolution MRI or histological verification.

On the other hand, the CT method has disadvantages as well. Since the voxel size currently is 88 microns, it makes electrode localization very challenging and in fact not very useful for localizing tetrodes and other thin structures with high enough resolution for most purposes. The authors should quantitatively compare advantages and disadvantages of their method. They should also emphasize that the CT method is not designed as a substitution of higher resolution histological method but as a preliminary screen. The detailed comments are available in the Reviewers' reports, which the authors should address carefully.

### Reviewer #1:

The present manuscript describes a method to register CT image of individual rat brains to MR rat brain atlas. This method is suggested as an alternative of classical histological procedures for localizing implanted electrodes. The proposed technique would help researchers to localize their multiple electrodes within a very short period of time (15 min) in contrast to the time and energy consuming conventional anatomical verification.

This is a good idea, however, I have a few concerns.

Major concerns:

1. The most problematic point of this method is the resolution of the micro CT image. The voxel size is 88 microns, which makes electrode localization very challenging (if not impossible) in small structures such as for example thalamic nuclei.
2. The accuracy of this co-registration is not optimal, mostly not in the anterior part of the brain, as the authors recommend visual verification of the registration (Discussion lines 341-352).

Overall, this method has two big advantages over classical histology, which is its low time and energy consumption, and the possibility of electrode localization in the living (but sleeping) animal. In my opinion, it cannot replace conventional anatomical verification of electrode tracks. Electrode localization in small structures is very uncertain due to the large voxel size combined with possible differences of individual rats/rat strains. Furthermore, the authors argue that slicing usually does not reach the optimal plane of the electrodes. This can be solved by reconstructing brain structure and the tracks of the implanted electrodes in 3D by for example the NeuroLucida system. It definitely requires more time and energy consumption than CT-MR co-registration, but it gives highly accurate result of electrode locations.

I would suggest the authors to rewrite the article in a way to emphasize the supplemental advantages of this method instead of suggesting it as a substitute for classical anatomical electrode track verification.

Minor concerns:

1. Artifact reduction shown on Figure 2B does not improve the original image. In contrast, it seems that electrodes on the artifact reduced image are larger than those on the original picture. I suggest to quantify electrode thickness on original pictures and on images with artifact reduction to verify its efficiency.
2. I do not understand the importance of Figure 1. The manuscript describes the transformation and co-registration of CT and MR images. Showing electrode and circuit board connections is inadequate and unnecessary.
3. Image transformation procedure described in Methods (Steps 1-4, lines 155-200) would be easier to understand if the different steps would be presented on a figure (instead of Figure 1 for example).
4. The authors do not describe the thickness of their implanted electrodes (Methods). Specify, please.
5. "88 mm isotropic voxel size" (line 155) should be 88 microns, I guess...
6....brain slices are frequently lost during the process of cutting or staining" (line 60)

This sentence should be reconsidered. A good anatomist (and even a good technician) does not lose any brain section during cutting and staining.

### Reviewer #2:

The authors present a method to localize the position of multiple electrodes implanted in the rat brain using uCT corregistered to an MRI template and atlas. The idea is not new and similar approaches and tools in humans have been already provided (i.e Azarion et al. Epilepsia 2014, Mirzadeh et al. Mov Disord 2014, Princich et al. Front Neurosci 2013). However, the presented work is of potential utility for the neuroscientific community and the ever growing number of reported rat/mice electrophysiological experiments with multiple recordings.

The work presents important limitations in order to evaluate its achievements and the utility of the proposed strategy. The manuscript is not scholarly written.

- The multi-step registration procedure described in the paper seems extremely data-dependent (and hardly reproducible). A figure-scheme describing the whole methodology would be useful to understand the presented work. What do the authors think about the reproducibility of the presented methodology? Which registration failure rate do they obtain? Which are the main sources of these failures?

This should be described in the paper, as this information is crucial for any potential readerThe major one is the lack of appropriate quantitative descriptions of the results.

- The authors say that "the electrode implant would have very high intensity values" (line 163), but they do not explain how is this signal distinguished from the bone's signal (which is also high).
- What is the source for the used MR images and how are they processed?
- In line 165 the authors talk about an "estimated probability density" for which they provide a value, however they do not explain how it is calculated.
- The manuscript offers a method which is fundamentally based on corregistering CT images to MRI to identify electrodes location. However, the algorithm used for the corresgistration is not explained. Only the name of a MatLab function and some parameters are provided (lines 186-187).
- When evaluation of corregistration comes to play, the authors refer to "visual inspection" (line 259) and conclude that they obtain "acceptable results (Fig. 4)"(line 262). Quantifications are required to demonstrate the quality of this critical step.
- In several places of the document the authors acknowledge problems in the corresgistration between uCT and MRI ("occasional registration failure" line 342, ...), but it is not quantified. How frequent are these failures?

Minor points:

- There are many typos in the text (i.e. line 27 "over dramatic" instead of "offer dramatic"?, line 155, mm instead of um,...)
- Reference to figures in the text is chaotic; Fig. 5 appears in line 212, before figures 2-4.
- Tables appear in the middle of the text without heading or legend
-Figures are poorly labeled (i.e Fig. 4, letters and numbers to refer to columns and rows are not a bad option after all...)

Dear Dr. Buzsaki,

Thank you for reviewing our manuscript "Localization of metal electrodes in the intact rat brain using registration of 3-D micro-computed tomography images to a magnetic resonance histology atlas." The Reviewers' comments were very insightful, and we have revised our manuscript to reflect their suggestions. We believe these changes have substantially improved the quality of our manuscript.

Below, please find a point-by-point reply to each of the Reviewer's concerns. All relevant or substantial revisions are highlighted in blue in the revised manuscript. We hope you will evaluate our revised submission positively for publication in eNeuro.

Sincerely,

The Authors

## Replies to Reviewer's Concerns

As suggested by Reviewer 1, we have revised the paper to emphasize that our digital imaging method is best used as a supplement to classical anatomical electrode track verification, rather than a replacement. We also learned from the Reviewers' comments that our original manuscript did not make it clear how easy it is to identify and correct registration failures. The current manuscript provides more detail about how to correct registration failures, which we believe will assuage concerns about the reliability of the method.

### Reviewer 1:

1. The most problematic point of this method is the resolution of the micro CT image. The voxel size is 88 µm, which makes electrode localization very challenging (if not impossible) in small structures such as for example thalamic nuclei.
  We understand the reviewer's concern. Two things address that concern. First, we were limited to 88 µm due to the fact that our micro-CT system was designed for in vivo micro-CT applications where sampling time and radiation dosages are constraints. Commercial micro-CT systems are available with spatial resolutions as high as 4.5 µm (see, for example, the Quantum GX microCT Imaging System by PerkinElmer: http://www.perkinelmer.com/catalog/product/id/quanuct), but these higher resolutions require higher radiation doses and longer scanning times, and therefore are most appropriate for postmortem applications. The registration methodology we describe here would apply for images collected from this type of higher resolution CT system as well. This information is now included in the Discussion (p. 21).
  Second, a resolution of 88 µm is adequate for localization to many brain structures of interest to neuroscientists and physiologists, including the brain regions of interest here.
2. The accuracy of this co-registration is not optimal, mostly not in the anterior part of the brain, as the authors recommend visual verification of the registration (Discussion lines 341-352).
  We appreciate the Reviewer's insight. Our simulations did suggest that fiducials in the most anterior part of the brain were most likely to be affected by the change in registration parameters caused by the presence of electrodes. If registration is suboptimal, however, a quick visual inspection of the overlap between the skull of the MR atlas and the CT images will be sufficient to determine whether the registration can be improved. This procedure is now described in the "Assessment of Registration Success" section of the Methods (p.13-14). Registration can be improved by tuning the registration parameters. We have now given more details on how to tune these parameters in the Discussion section (p. 22).

### Reviewer 2:

3. The multi-step registration procedure described in the paper seems extremely data-dependent (and hardly reproducible). A figure-scheme describing the whole methodology would be useful to understand the presented work. What do the authors think about the reproducibility of the presented methodology? Which registration failure rate do they obtain? Which are the main sources of these failures? This should be described in the paper, as this information is crucial for any potential reader. The major one is the lack of appropriate quantitative descriptions of the results.
  The suggestion to add a figure depicting the registration procedure was very helpful. Figure 2 now includes a schematic of the entire micro-CT to MRH registration procedure. More background information about the general methodology illustrated in this schematic is now provided in the Methods section (pp. 8-9). This background clarifies why our procedure will apply to other micro-CT to MRH registration projects, and other types of electrode implantation procedures. We have provided more detail about how we assessed the performance of our procedure in the Methods section (pp. 13-14), and added more details about the results of this assessment in the Results section (pp. 17-18). We also provide quantification of our failure rate due to electrode configuration in Figure 6 (formerly Figure 5). We have now given more details about how to identify and correct failures in the Methods (pp. 13-14), Results (p. 19), and Discussion section (p. 22).

### Reviewer 1:

1. Artifact reduction shown on Figure 2B does not improve the original image. In contrast, it seems that electrodes on the artifact reduced image are larger than those on the original picture. I suggest to quantify electrode thickness on original pictures and on images with artifact reduction to verify its efficiency.
  CT images are affected by metallic artifacts that cannot be completely eliminated using any currently available correction method. Metal causes beam hardening and scatter effects, which result in dark streaks between metal objects, and surrounding bright streaks. These streaks impair registration, which is why we recommend masking out the artifacts as much as possible at key parts of the registration procedure. The correction is necessary for successful registration even when these masking steps are implemented, however. We have examined how the correction affects the size of the electrode bundles and have determined that while it does make the electrodes appear thicker (by 1-2 voxels on each side of an electrode), it rarely makes them appear longer (and if it does make them appear longer, it does so by no more than one faint voxel). Since the central axes of the electrode bundles remain the same after correction, the central axes can be used to determine the lateral and posterior/anterior location of bundles without affecting the electrode localization accuracy.
2. I do not understand the importance of Figure 1. The manuscript describes the transformation and co-registration of CT and MR images. Showing electrode and circuit board connections is inadequate and unnecessary.
  We apologize for the lack of clarity. The main purpose of Figure 1 was to allow readers with expertise in CT and MR imaging to appreciate the amount of metal in the electrode implant. These pictures make it clear why MR imaging is unsuitable, and help give a feel for the artifacts likely to appear in the CT images. .
3. Image transformation procedure described in Methods (Steps 1-4, lines 155-200) would be easier to understand if the different steps would be presented on a figure (instead of Figure 1 for example).
  Figure 2 now illustrates a schematic of the entire micro-CT to MRH registration procedure.
4. The authors do not describe the thickness of their implanted electrodes (Methods). Specify, please.
  Our electrodes were 50 µm thick, as now stated in the Methods on p. 6.
5. "88 mm isotropic voxel size" (line 155) should be 88 µm, I guess...
  Thank you for catching this typo. We have corrected it.
6. "brain slices are frequently lost during the process of cutting or staining" (line 60)
  This sentence should be reconsidered. A good anatomist (and even a good technician) does not lose any brain section during cutting and staining.
  We have now tempered this sentence to read "brain slices can be lost during the process of cutting or staining."

### Reviewer 2:

1. The authors present a method to localize the position of multiple electrodes implanted in the rat brain using uCT corregistered to an MRI template and atlas. The idea is not new and similar approaches and tools in humans have been already provided (i.e Azarion et al. Epilepsia 2014, Mirzadeh et al. Mov Disord 2014, Princich et al. Front Neurosci 2013).
  There is an important difference between the challenge our method addresses and the challenge addressed by the studies Reviewer 2 cites. The type of metal used in standard rodent electrophysiological studies makes it impossible to acquire MR images of each individual rat with electrodes already implanted. In contrast, the deep brain stimulation materials used in the human studies Reviewer 2 cites are MR-compatible. Thus, the registration process for the present application had to both cross modalities (CT to MR) and individual specimens (an individual CT scan to an atlas representing the average of 5 other specimens), whereas previous studies only needed to employ registration procedures that crossed modalities. We have clarified this in the current manuscript, and cited the studies Reviewer 2 references (p. 8).
2. The authors say that "the electrode implant would have very high intensity values" (line 163), but they do not explain how is this signal distinguished from the bone's signal (which is also high).
  We have now revised the text to read: "The method we used to isolate the electrode implant from the CT images in our preparation was based on the observation that voxels representing the electrode implant had higher intensity values than anything else in the image (including the skull) and appeared with a very low probability." (p. 11)
3. What is the source for the used MR images and how are they processed?
  The MR images were downloaded from the publically available Wistar Rat atlas we cited (Johnson et al, 2012). The acquisition methods and processing technique were described in detail in the cited reference. We have highlighted this fact in Step 1 of the "CT-to-MR Atlas registration" section of the revised manuscript (p. 10).
4. In line 165 the authors talk about an "estimated probability density" for which they provide a value, however they do not explain how it is calculated.
  The referenced text now reads: "Taking advantage of these features, the most straight-forward way to mask out the electrode implant was to estimate a probability density function of the intensity values in each of the CT image volumes. The estimate of this function was computed using the ksdensity function in Matlab (Mathworks, 2013), which is based on a normal kernel function and is evaluated at 100 equally spaced points that cover the full range of the inputted data." (p. 11)
5. The manuscript offers a method which is fundamentally based on corregistering CT images to MRI to identify electrodes location. However, the algorithm used for the corresgistration is not explained. Only the name of a MatLab function and some parameters are provided (lines 186-187).
  We have expanded our description of registration procedures and the specific algorithm we used throughout the Methods section of the current manuscript.
6. When evaluation of corregistration comes to play, the authors refer to "visual inspection" (line 259) and conclude that they obtain "acceptable results (Fig. 4)"(line 262). Quantifications are required to demonstrate the quality of this critical step.
  We have now expanded on the rationale for using visual inspection, and had 3 rat anatomy experts rate the success of the registration procedures on all seven rats (pp. 13-14).
7. In several places of the document the authors acknowledge problems in the corresgistration between uCT and MRI ("occasional registration failure" line 342, ...), but it is not quantified. How frequent are these failures?
  Failures are quantified in Figure 5 (now Figure 6). As discussed earlier, we have expanded our discussion of these failures in the main text, and expanded on how they would be identified and corrected.
8. There are many typos in the text (i.e. line 27 "over dramatic" instead of "offer dramatic"?, line 155, mm instead of um,...)
  Thank you for catching these typos. We have corrected them.
9. Reference to figures in the text is chaotic; Fig. 5 appears in line 212, before figures 2-4.
  We have ensured the figure references appear in chronological order.
10. Tables appear in the middle of the text without heading or legend
  We have added headings to the electrode table on p. 6 and the statistics table on p. 16.
11. Figures are poorly labeled (i.e Fig. 4, letters and numbers to refer to columns and rows are not a bad option after all...)
  We have added letters and numbers to the rows and columns in Figure 4 (now Figure 5).
